# Efficient production of myo-inositol in *Escherichia coli* through metabolic engineering

**DOI:** 10.1186/s12934-020-01366-5

**Published:** 2020-05-24

**Authors:** Ran You, Lei Wang, Congrong Shi, Hao Chen, Shasha Zhang, Meirong Hu, Yong Tao

**Affiliations:** 1 School of Life Sciences, University of Science and Technology of China, Hefei, 230027 China; 2 Chinese Academy of Sciences Key Laboratory of Microbial Physiological and Metabolic Engineering, Institute of Microbiology, Chinese Academy of Sciences, Beijing, 100101 China; 3 College of Life Science, University of Chinese Academy of Sciences, Beijing, 100049 China

**Keywords:** Metabolic engineering, *Escherichia coli*, Myo-inositol, High stoichiometric yield, High density fermentation, Bioconversion

## Abstract

**Background:**

The biosynthesis of high value-added compounds using metabolically engineered strains has received wide attention in recent years. Myo-inositol (inositol), an important compound in the pharmaceutics, cosmetics and food industries, is usually produced from phytate via a harsh set of chemical reactions. Recombinant *Escherichia coli* strains have been constructed by metabolic engineering strategies to produce inositol, but with a low yield. The proper distribution of carbon flux between cell growth and inositol production is a major challenge for constructing an efficient inositol-synthesis pathway in bacteria. Construction of metabolically engineered *E. coli* strains with high stoichiometric yield of inositol is desirable.

**Results:**

In the present study, we designed an inositol-synthesis pathway from glucose with a theoretical stoichiometric yield of 1 mol inositol/mol glucose. Recombinant *E. coli* strains with high stoichiometric yield (> 0.7 mol inositol/mol glucose) were obtained. Inositol was successfully biosynthesized after introducing two crucial enzymes: inositol-3-phosphate synthase (IPS) from *Trypanosoma brucei*, and inositol monophosphatase (IMP) from *E. coli*. Based on starting strains *E. coli* BW25113 (wild-type) and SG104 (*ΔptsG::glk*, *ΔgalR::zglf*, *ΔpoxB::acs*), a series of engineered strains for inositol production was constructed by deleting the key genes *pgi*, *pfkA* and *pykF*. Plasmid-based expression systems for IPS and IMP were optimized, and expression of the gene *zwf* was regulated to enhance the stoichiometric yield of inositol. The highest stoichiometric yield (0.96 mol inositol/mol glucose) was achieved from recombinant strain R15 (SG104, *Δpgi*, *Δpgm*, and RBSL5-zwf). Strain R04 (SG104 and *Δpgi*) reached high-density in a 1-L fermenter when using glucose and glycerol as a mixed carbon source. In scaled-up fed-batch bioconversion in situ using strain R04, 0.82 mol inositol/mol glucose was produced within 23 h, corresponding to a titer of 106.3 g/L (590.5 mM) inositol.

**Conclusions:**

The biosynthesis of inositol from glucose in recombinant *E. coli* was optimized by metabolic engineering strategies. The metabolically engineered *E. coli* strains represent a promising method for future inositol production. This study provides an essential reference to obtain a suitable distribution of carbon flux between glycolysis and inositol synthesis.

## Background

Myo-inositol (inositol), a water-soluble vitamin B group compound with a six-carbon ring, is widely used in many industries such as food, pharmaceuticals and cosmetics [1–4]. Inositol is also a precursor of various biofunctional compounds containing inositol phosphate and lipid [5, 6]. Inositol has attracted the attention of the food and feed industries, where it is mainly used as an additive in drinks, in milk powder as a nutritional supplement, and in animal feed to prevent diseases [7, 8]. Inositol has been proved to treat some diseases such as diabetes and fatty liver [9–11]. Furthermore, inositol is an important intermediate used for the production of uronic acid and inositol derivatives. There are a few reports describing the use of whole-cell biocatalysis to produce glucuronic acid, glucaric acid and scyllo-inositol from inositol or glucose [12–17].

Microbial production of inositol is regarded as a promising alternative to conventional methods of synthesis such as hydrolysis of phytate [18, 19]. The synthesis pathway of inositol from the precursor glucose-6-phosphate (G-6-P) involves two crucial enzymes, inositol-3-phosphate synthase (IPS) and inositol monophosphatase (IMP). Research applying G-6-P to produce inositol via a two-step reaction has been extensive. IPS, the key rate-limiting enzyme in biosynthesis of inositol, can convert G-6-P to the intermediate inositol-3-phosphate (inositol-3-P). The final product inositol is reached by IMP-catalyzed dephosphorylation of inositol-3-P [20–22]. There are some reports concerning in vitro enzymatic production of inositol using G-6-P, and G-6-P was obtained from starch through two enzymes, alpha-glucan (or maltodextrin) phosphorylase and phosphoglucomutase [21, 22]. We have previously reported a trienzymatic cascade system of polyphosphate glucokinase, IPS and IMP to produce inositol from glucose, giving a molar conversion of 90% [20]. However, enzymatic production of inositol had many problems, including a complicated process of isolation, high cost, and instability of enzymes [23]. Inositol has been produced from glucose by using metabolically engineered *Escherichia coli* strains. Nevertheless, the yield was low, mainly because of the competitive relationship between cell growth and inositol synthesis [24]. The low yield indicates that only a small amount of G-6-P is distributed to inositol production. Therefore, an inositol biosynthesis pathway in recombinant *E. coli* strains needs to be metabolically optimized. Our laboratory has developed an *E. coli* strain (SG104) in which the glucose utilization system was modified by replacing native *ptsG* (gene ID: 945651) and *galR* (gene ID: 947314) with *glk* (gene ID: 946858) and *zglf* (galactose:H^+^ symporter from *Zymomonas mobilis*) respectively in the chromosome of *E. coli* BW25113, and the accumulation of acetic acid was reduced by deleting *poxB* (gene ID: 946132) and overexpressing *acs* (gene ID: 948572) for high-density fermentation [25].

**Fig. 1:**
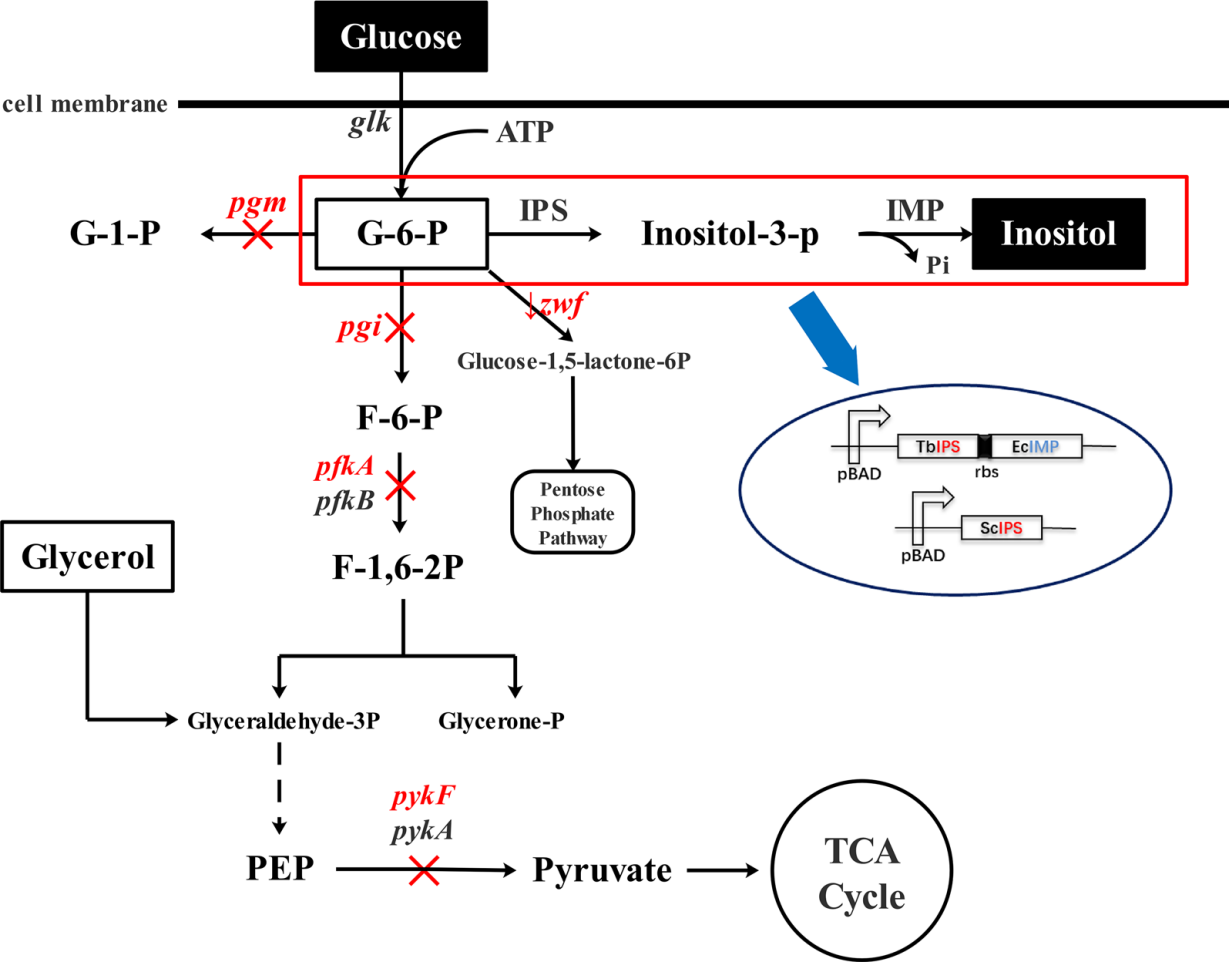
Overview of the inositol biosynthesis pathway in *Escherichia coli*

In the present study, our aim was to achieve high stoichiometric yields of inositol, by guiding the carbon flux of precursor G-6-P to inositol synthesis. A series of metabolic engineering strategies was performed in the inositol synthesis pathway (Fig. 1). First, a biosynthesis pathway for inositol from G-6-P was designed by overexpressing two crucial enzymes—IPS and IMP—and the plasmid expression systems of IPS and IMP were optimized. Second, to enhance metabolic flux to G-6-P, *pgi* (G-6-P isomerase, gene ID: 948535), *pfkA* (6-phosphofructokinase I, gene ID: 948412), *pykF* (pyruvate kinase II, gene ID: 946179), and *pgm* (phosphoglucomutase, gene ID: 945271) were deleted. Thereafter, the gene *zwf* (gene ID: 946370), encoding G-6-P dehydrogenase in the pentose phosphate pathway (PPP), was regulated by replacement of the promoter or ribosome binding site (RBS) to further improve inositol production. The highest stoichiometric yield (0.96 mol inositol/mol glucose) was obtained from strain R15 (SG104, *Δpgi*, *Δpgm*, RBSL5-zwf). Third, four recombinant strains (R04, R12, R14 and R15) with high stoichiometric yields (> 0.7 mol inositol/mol glucose) were chosen for high-density fermentation using glucose and glycerol as a mixed carbon source. Finally, strain R04 (SG104 and *Δpgi*) reached the density OD_600_ = 135 after 48 h, and scaled-up production of inositol through bioconversion in situ was accomplished to obtain a titer of 106.3 g/L (590.5 mM), corresponding to a stoichiometric yield of 0.82 mol inositol/mol glucose.

## Results

### Production of inositol by slowing carbon flux in glycolysis pathway

The intake of glucose in *E. coli* involves two key enzymes: glucose kinase (*glk*) and protein-Npi-phosphohistidine-d-glucose phosphotransferase (*ptsG*). Glucose kinase converts glucose to G-6-P with high catalytic efficiency [26–28]. Our laboratory has developed an *E. coli* strain, SG104, by deleting *ptsG* and enhancing *glk* to increase glucose intake [25]. *E. coli* BW25113 and SG104 were chosen as starting strains to construct host strains that slow carbon flux to glycolysis to enhance the supply of inositol precursor G-6-P. The key genes involved—*pgi*, *pfkA* and *pykF*—were respectively deleted (Fig. 1). Deletion of *pgi* directly enhances precursor G-6-P accumulation by blocking glycolysis. The *pfkA* redirected carbon flux in glycolysis pathway, and showed most of 6-phosphofructokinase activity [24]. Previous study has shown that *pykF*, the major pyruvate kinase, is a regulatory factor of glycolysis [29]. Our previous study indicated that TbIPS from *Trypanosoma brucei* and EcIMP from *E. coli* have high specific activity [20]. As such, plasmid p01 expressing TbIPS and p02 expressing EcIMP were co-transformed into *E. coli* strains BW25113, SG104, R01, R02, R03, R04, R05 and R06 respectively to construct recombinant strains for inositol production.

**Fig. 2:**
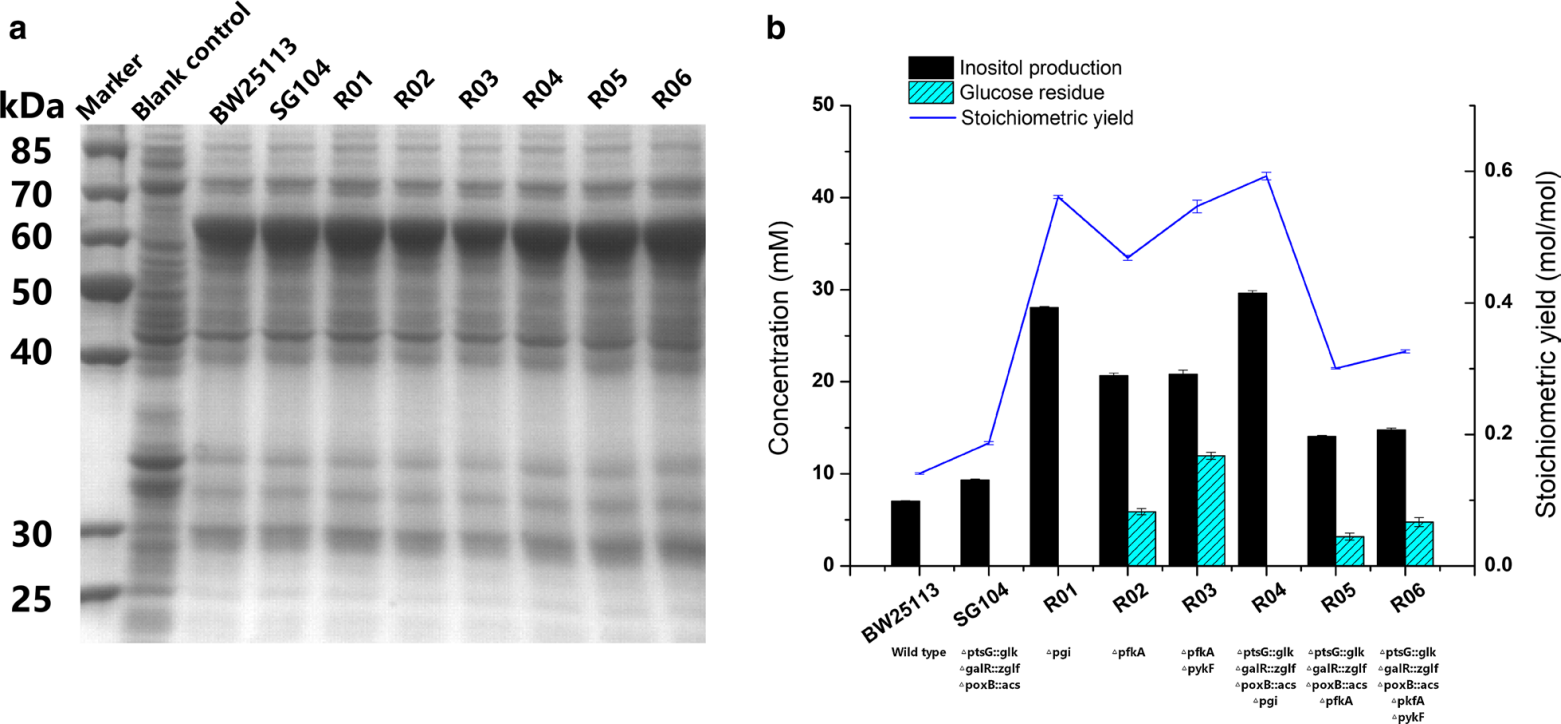
Host strain selection. **a** SDS-PAGE showing the expression of inositol-3-phosphate synthase (IPS; approximately 60 kDa) and inositol monophosphatase (IMP; approximately 30 kDa) in different chassis strains. **b** Production of inositol in different chassis strains. The recombinant strains transformed with plasmids p01 and p02 were induced and then suspended in a bioconversion mixture containing 50 mM glucose. The bioconversions were performed for 10 h at 37 °C and 220 rpm

The expression of IPS and IMP in different host strains was shown as Fig. 2a. Whole-cell bioconversions were performed using different strains to select an appropriate host strain for inositol production. After 10 h of bioconversion, 29.6 mM inositol was obtained using strain R04; a stoichiometric yield of 0.6 mol inositol/mol glucose was reached, and no residual glucose was observed (Fig. 2b). Compared with strain R01 in which *pgi* is deleted to block glycolysis, strain R04 derived from strain SG104 showed increased inositol production. The results showed that deletion of *pgi* was effective for accumulation of precursor G-6-P. Strains R05 and R06 showed faster glucose consumption than R02 and R03, but low inositol concentration was achieved (Fig. 2b).

### Improvement of inositol production by optimizing plasmid expression systems

**Fig. 3:**
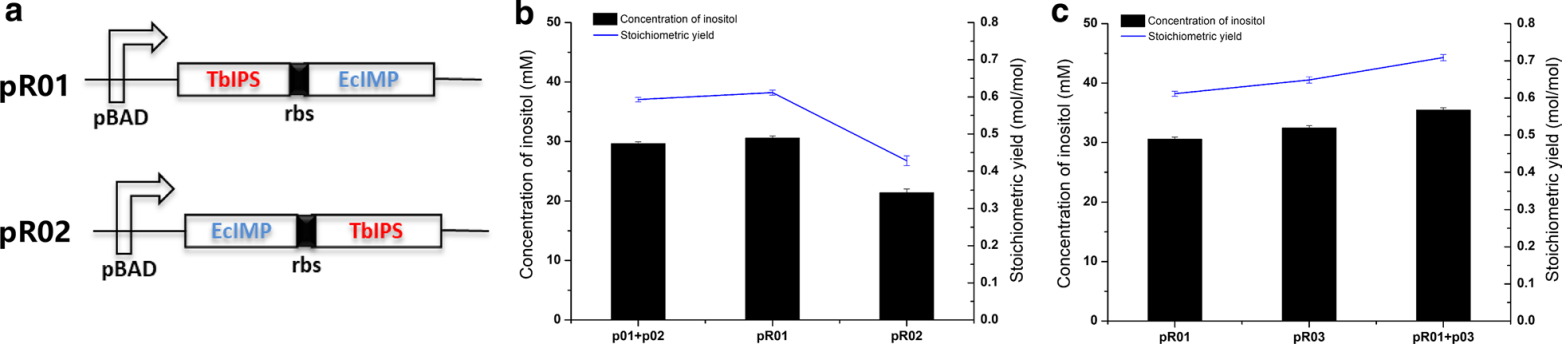
Effects of different plasmid combinations in host strain R04 on inositol production. **a** Schematic diagram of pR01 and pR02 in which the genes encoding TbIPS and EcIMP are arranged in different sequences. rbs, ribosome binding site; pBAD, an araBAD promoter. **b** Inositol production using different plasmids singly or in combination. **c** Effect of IPS expression on introducing ScIPS from *Saccharomyces cerevisiae*

Plasmid expression systems are useful for the reconstruction of biosynthesis pathways and usually give a high yield of a target product [25]. To investigate the effect of expression of TbIPS and EcIMP from a single plasmid, plasmids pR01 and pR02 were constructed (Fig. 3a) and respectively transformed into host strain R04 to produce inositol. The results showed that pR01 improved the production of inositol; 0.62 mol inositol/mol glucose was produced with a titer of 31.1 mM (Fig. 3b). The expression of key enzymes (TbIPS and EcIMP) is shown in Additional file 1: Fig. S1a.

The activity of IPS in the metabolic pathway is one of the most important factors [20]. Therefore, ScIPS from *Saccharomyces cerevisiae* was introduced to enhance IPS activity in the inositol-biosynthesis pathway. Plasmids pR03 and p03 were constructed, and plasmid combinations pR01 + p03, and pR03 were respectively transformed into host strain R04 to produce inositol. The cells transformed with pR01 + p03 produced 0.71 mol inositol/mol glucose with a titer of 35.5 mM (Fig. 3c). The expression of TbIPS, ScIPS, and EcIMP is shown in Additional file 1: Fig. S1b.

### Further strain optimization by regulating *zwf* and deleting *pgm*

To further enhance the stoichiometric yield of inositol, the strength of expression of gene *zwf* from the PPP was adjusted by replacement of its promoter or RBS. Strain R04 was chosen as the platform strain to construct seven host strains (R7 to R13), which were used as controls to evaluate the effects of blocking and enhancing the PPP, respectively. Expression of *zwf* was decreased in strains R08 to R12 by using different RBS strengths (RBSL1 to RBSL5).

**Fig. 4:**
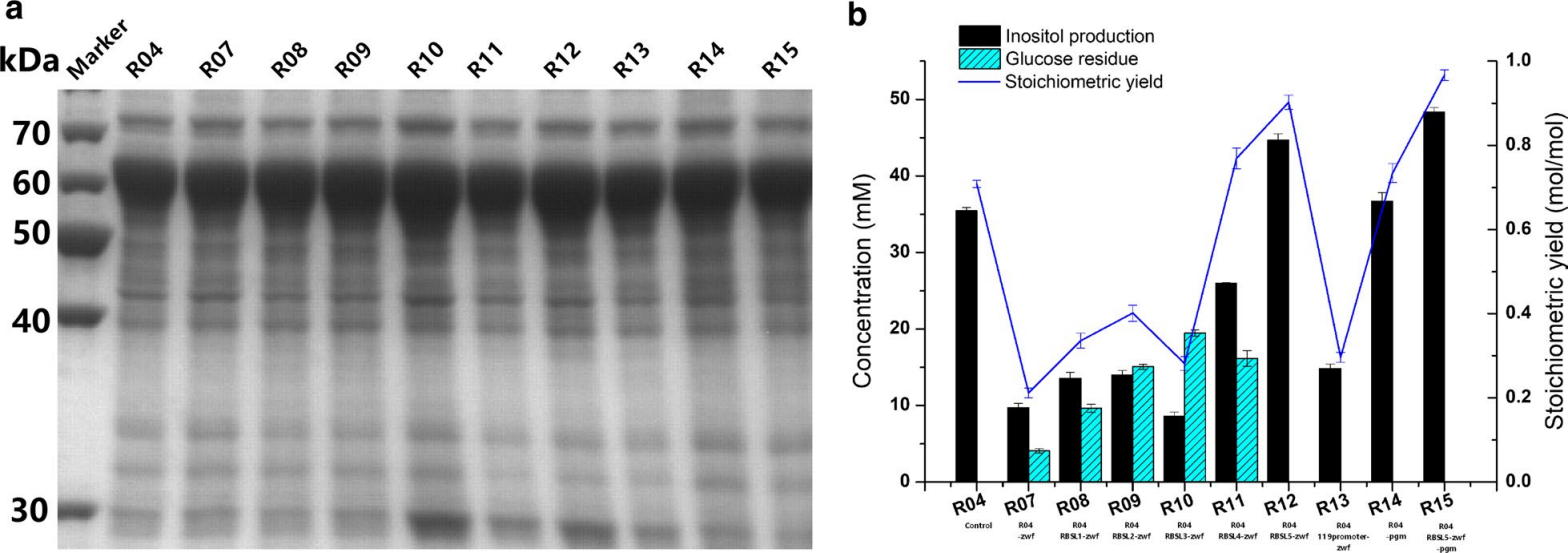
Effects of regulating *zwf* and deleting *pgm* on inositol production. **a** SDS-PAGE showing the expression of IPS (approximately 60 kDa) and IMP (approximately 30 kDa). **b** Production of inositol in different host strains. The recombinant strains were induced and harvested, then suspended in a bioconversion mixture containing 50 mM glucose. The bioconversions were performed at 37 °C and 220 rpm for 10 h

The resulting host strains were then transformed with the plasmid combination pR01 + p03. The expression of key enzymes (TbIPS, ScIPS, and EcIMP) in host strains (R04, and R07 to R15) is shown in Fig. 4a. Strain R12 exhibited inositol production of 44.7 mM after 10 h of bioconversion, corresponding to a stoichiometric yield of 0.9 mol inositol/mol glucose (Fig. 4b). The gene *pgm*, encoding phosphoglucomutase, which acts in isomerization of G-6-P and glucose-1-phosphate, was deleted to construct host strains R14 and R15. The highest stoichiometric yield (0.96 mol inositol/mol glucose) was achieved in strain R15, corresponding to a concentration of 48 mM inositol and consumption of 50 mM glucose (Fig. 4b).

### High-density fermentation through synergetic utilization of glucose and glycerol

To evaluate the fermentation capacity of recombinant strains, host strains R04, R12, R14 and R15 transformed with plasmids pR01 + p03 were chosen for high-density fermentation using a modified inorganic salts medium containing glucose and glycerol. The stoichiometric yields produced by the four strains in shaken flasks were all > 0.7 mol inositol/mol glucose (Fig. 4b). *E. coli* follows the glycolysis pathway as the central metabolic system for cell growth. However, glycolysis was blocked by deletion of *pgi*; thus glycerol was selected as a carbon source to enable cell growth.

**Fig. 5:**
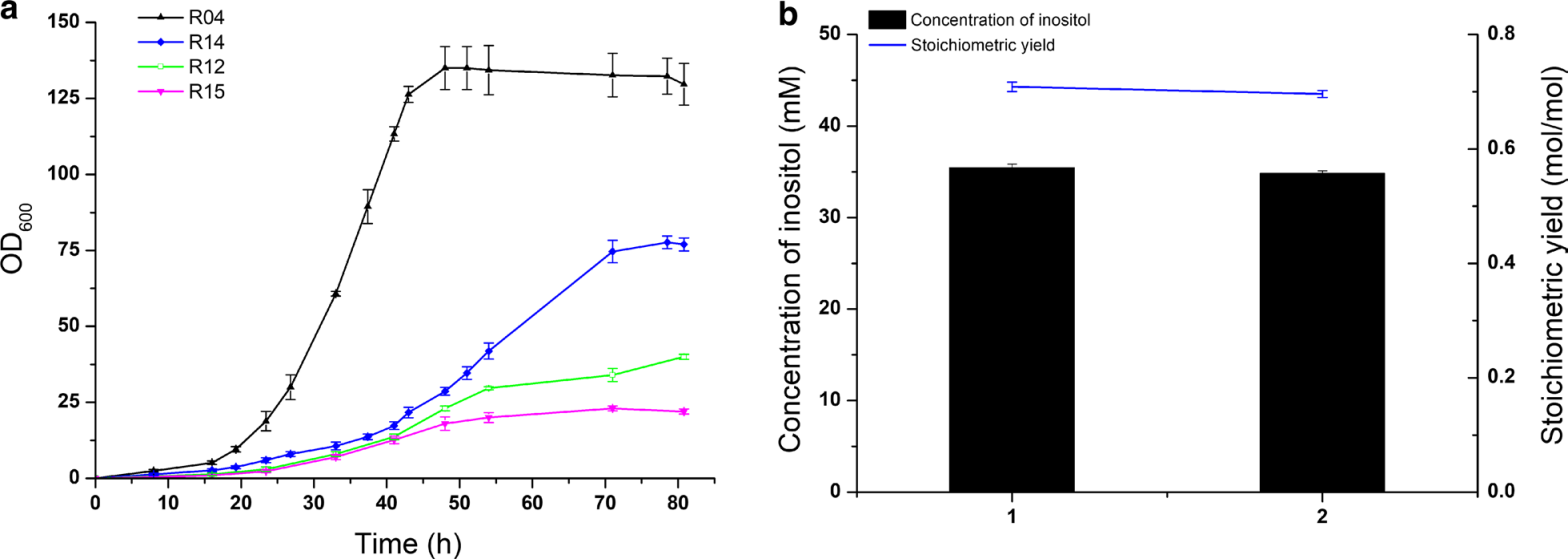
High-density fermentation of recombinant strains with high stoichiometric yields and bioconversion by strain R04. **a** High-density fermentation of strains R04, R12, R14, and R15. Cells were initially cultured in Luria–Bertani medium at 37 °C. Then, this seed culture was inoculated into 500 mL inorganic salts medium with glycerol and glucose as a mixed carbon source in a 1-L fermenter. **b** Comparison of bioconversion by strain R04 cultured in shaken flasks (1) and high-density fermentation (2). Cells were suspended in a mixture with 50 mM glucose for 10 h at 37 °C and 220 rpm

The four recombinant strains were cultivated in modified inorganic salts medium with glycerol and glucose as a mixed carbon source. Strain R04 reached a density of OD_600_ = 135 after 48 h. However, the other three strains could not grow, or grew slowly, in our modified inorganic salts medium (Fig. 5a). The expression of key enzymes (TbIPS, ScIPS, and EcIMP) in strain R04 in the high-density fermentation is shown in Additional file 1: Fig. S2. In both shaken flasks and high-density fermentation, strain R04 showed approximately equivalent stoichiometric yields (> 0.7 mol inositol/mol glucose) without glucose residue (Fig. 5b). The other three strains (R12, R14 and R15) showed poor expression of key enzymes and low stoichiometric inositol yield (Additional file 1: Fig. S3). The results indicated that the high-density fermentation of strain R04 could be improved through synergetic use of glucose and glycerol as carbon sources.

### Scaled-up production of inositol using strain R04

**Fig. 6:**
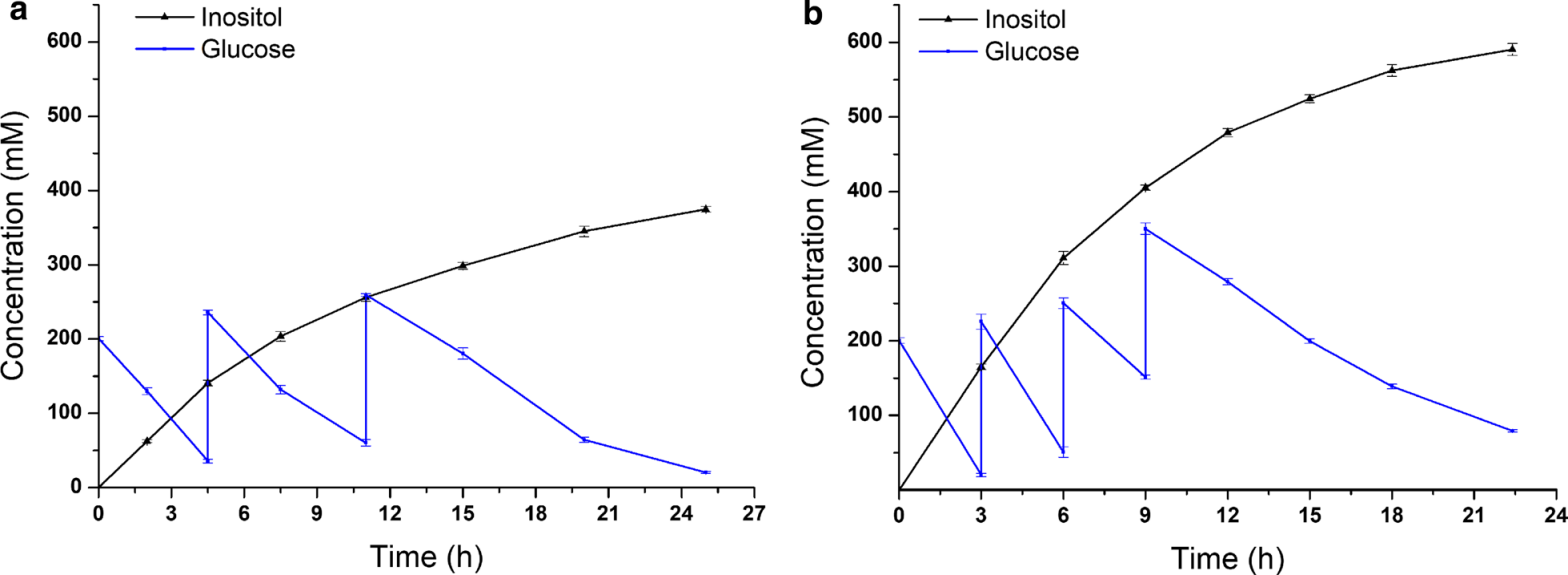
Scaled-up production of inositol by bioconversion by strain R04 ex situ (**a**) and in situ (**b**). The bioconversion ex situ was in a 1-L fermenter containing biomass (OD_600_ = 80), 1 mM MgSO_4_, and 1 × M9 salts buffer. Glucose was added in batches; 200 mM glucose was added at 0, 4.5, and 11 h respectively. The bioconversion in situ in a 1-L fermenter was started when the cells grew to the density of OD_600_ = 80. Glucose was added in batches; 200 mM glucose was added at 0, 3, 6, and 9 h, respectively

Strain R04 reached high-density through synergetic use of glucose and glycerol. To comprehensively evaluate the overall production performance of inositol, scaled-up bioconversion using strain R04 was carried out in a 1-L fed-batch fermenter. After 25 h of bioconversion ex situ, 375 mM inositol was obtained, while 580 mM glucose was consumed. The stoichiometric yield was 0.65 mol inositol/mol glucose (Fig. 6a). 0.82 mol inositol/mol glucose was produced after 23 h of bioconversion in situ, while 720.5 mM glucose was consumed. The concentration of inositol reached 590.5 mM, corresponding to a titer of 106.3 g/L (Fig. 6b).

## Discussion

Inositol and its derivatives are widely distributed in nature, displaying a variety of biological activities and metabolic functions; they are also valuable as nutrients and pharmaceuticals [30–32]. The tools of metabolic engineering and synthetic biology have been combined in recent years to design microorganisms with desirable functions. This has motivated many studies to examine the production of high-value products from glucose, which is cheap [33–35]. Efficient production of inositol via biotechnological approaches is urgently needed. *E. coli* is considered a “generally regarded as safe” organism and possesses a glycolysis pathway that synthesizes the inositol precursor G-6-P. Inositol has been produced from glucose by using metabolically engineered *E. coli*, but low yields have restricted industrial production of inositol by this method [24]. Metabolic engineering of *E. coli* to efficiently supply G-6-P would enhance the production of inositol.

Numerous studies have explored various approaches for reallocating the metabolic flux in *E. coli* to attenuate glycolysis and accumulate G-6-P [24, 29]. In this study, the key targets *pgi*, *pfkA*, *pykF*, and *pgm* were deleted to enhance the supply of G-6-P. Thereafter, the key gene *zwf* in the PPP was modified by replacement of its promoter or RBS to further accumulate G-6-P (Fig. 1). It is not surprising that this study found evidence that the accumulation of precursor G-6-P was beneficial for production of inositol. This was substantiated by the significant increase in stoichiometric yield observed upon knockout of *pgi* (Fig. 2). In addition, the intake of glucose is also a key issue to be addressed, and the SG104 background was also benefit inositol production (Additional file 1: Fig. S4a). Furthermore, the SG104 background promoted cell growth in high-density fermentation (Additional file 1: Fig. S4b). The activity of IPS is important to the effective synthesis of inositol from G-6-P. Increasing the activity of IPS by introducing two such enzymes from different sources (i.e., TbIPS and ScIPS) showed great potential for boosting the production of inositol. In the present inositol biosynthesis pathway, 1 mol of glucose is, in principle, metabolized to 1 mol of inositol via a three-step reaction. It is important to design the metabolic pathway toward the compound(s) of interest by considering the stoichiometric yield. The highest yield in our study was 0.96 mol inositol/mol glucose in strain R15, approaching the theoretical stoichiometric yield (Fig. 4b).

Suitable distribution of carbon flux between cell growth and inositol synthesis is the second challenge to be addressed for inositol production from glucose using microbial cell factories. However, here, the growth of recombinant strains was restricted because glycolysis was blocked to supply G-6-P. The strains in which *pgi* is deleted almost could not grow in organic salts *(*i.e., M9 salts) medium with glucose. This seriously impedes the efficient production of inositol. Glycerol is a valuable carbon source for cell growth in microbial fermentation. Hence, the use of glucose and glycerol as a mixed carbon source may be a promising strategy for high-density fermentation. In our study, four recombinant strains (R4, R12, R14, and R15) were cultivated in modified inorganic salts medium containing glucose and glycerol as a mixed carbon source. Disappointingly, only strain R04 reached high-density (OD_600_ = 135), while the growth of strains R12, R14, R15 was poor (Fig. 5a). Ex situ and in situ bioconversions for inositol production by strain R04 were carried out in a 1-L fed-batch fermenter. A high stoichiometric yield (0.82 mol inositol/mol glucose) was achieved, and 720.5 mM glucose was consumed, corresponding to production of 590.5 mM inositol (Fig. 6b). The titer of inositol, 106.3 g/L, was higher than that in most previous reports, and is promising for scaling up for commercial applications. Balancing cell growth and inositol production is expected to be a powerful strategy for industrial production of inositol.

Besides metabolic engineering approaches, optimization of cultivation conditions and bioconversion processes will be important to improve inositol production. Considering that appropriate coupling between cell growth and inositol synthesis is critical for the overall inositol production, high-density fermentation could be further optimized to enhance inositol yield. It is also generally accepted that recombinant strains (strain R12, R14, R15) should be considered to evolve.

## Conclusions

In the present study, we performed inositol production using different engineering strategies to construct *E. coli* host strains. Enhancement of the supply of precursor G-6-P in the inositol-biosynthesis pathway, and optimization of high-density fermentation by using glucose and glycerol as a mixed carbon source, were key strategies to enhance inositol production. The combination of these approaches increased the stoichiometric yield to 0.96 mol inositol/mol glucose. Glycolysis in *E. coli* was blocked by inactivating the gene *pgi* to redirect the carbon flux toward inositol production, but this resulted in restriction of cell growth. Fortunately, recombinant strain R04 reached high-density through synergetic use of glucose and glycerol. Bioconversions for inositol production were carried out ex situ and in situ in a 1-L fermenter by using cells at high density. Finally, we achieved 590.5 mM inositol (a titer of 106 g/L) by using the bioconversion in situ, corresponding to a stoichiometric yield of 0.82 mol inositol/mol glucose. The coupling of high-density fermentation of engineered strains and bioconversion in situ may therefore be a promising strategy for efficient production of inositol with high stoichiometric yield and titer.

## Methods

### Chemicals and media

T4 DNA ligase, Gibson kits and restriction enzymes were purchased from New England Biolabs (USA). Plasmid extraction and gel purification kits were purchased from Omega (Beijing, China). DNA polymerase (2× High Fidelity Master Mix) was bought from TSINGKE (Beijing, China). Media components were bought from Becton–Dickinson (Beijing, China). Standards of Glucose, G-6-P, inositol, and other chemicals were obtained from Sigma-Aldrich (Shanghai, China).

All *E. coli* strains were grown in Luria–Bertani (LB) medium containing 10 g/L tryptone, 10 g/L NaCl, and 5 g/L yeast extract. Modified inorganic salts medium was used for high-density fermentation of engineered strains. Seed culture was inoculated into modified inorganic salts medium containing mixed carbon sources (10 g/L glucose and 10 g/L glycerol), KH_2_PO_4_ 14 g/L, (NH_4_)_2_PO_4_ 4 g/L, Na_2_SO_4_ 2 g/L, citric acid 1.8 g/L, MgSO_4_·7H_2_O 0.6 g/L, and trace elements [8.4 mg/L EDTA, 2.5 mg/L CoCl_2_·6H_2_O, 0.15 mg/L MnCl_2_·4H_2_O, 1.5 mg/L CuCl_2_·2H_2_O, 3 mg/L H_3_BO_3_, 2.5 mg/L Na_2_MoO_4_·2H_2_O, 13 mg/L Zn(CH_3_COO)_2_·2H_2_O, and 10 g/L Fe^3+^citrate]. The feeding medium contained 600 g/L mixed carbon sources, 2 g/L MgSO_4_·7H_2_O, and trace elements.

### Strains and plasmids

The bacterial strains and plasmids used in this study are listed in Table 1. Primers used in the study are listed in Additional file 1: Table S1. The plasmids pYB1s and pLB1a used for expression of genes are derived from our laboratory’s vectors, which have the origin of replication, streptomycin and ampicillin resistance genes, an araBAD promoter (pBAD), multiple cloning sites, and a *rrnB* terminator. The IPS genes from *T. brucei* and *S. cerevisiae* were codon-optimized and synthesized by General Biosystems Co., Ltd. (Anhui, China). The encoding nucleotide sequences of the enzymes used for producing inositol were amplified by PCR and ligated into the vectors between *Xho*I and *Spe*I sites by T4 ligation and Gibson assembly [36]. *E. coli* transT1 was used for molecular cloning. *E. coli* BW25113 was used as the parental strain for genetic modification and inositol production. Gene knockout strains were obtained from the KEIO collection (National BioResource Project) [37, 38]. P1 virus-mediated transfection was used for integration into the chromosome [39, 40]. The Crispr-Cas9 system was used for gene knockout, replacement, and change of RBS or promoter [41].

**Table 1:** Strains and plasmids used in this study

Plasmid/strain	Description	References
Strains		
*E. coli* transT1	Wild-type	Invitrogen
*E. coli* BW25113	Wild-type	Invitrogen
SG104	*E. coli* BW25113,Δ*ptsG*::*glk,*Δ*galR*::*zglf,*Δ*poxB*::*acs*	Laboratory
R01	*E. coli* BW25113,Δ*pgi*	This study
R02	*E. coli* BW25113,Δ*pfkA*	This study
R03	*E. coli* BW25113,Δ*pfkA,*Δ*pykF*	This study
R04	*E. coli* BW25113,Δ*ptsG*::*glk,*Δ*galR*::*zglf,*Δ*poxB*::*acs*,Δ*pgi*	This study
R05	*E. coli* BW25113,Δ*ptsG*::*glk,*Δ*galR*::*zglf,*Δ*poxB*::*acs*,Δ*pfkA*	This study
R06	*E. coli* BW25113,Δ*ptsG*::*glk,*Δ*galR*::*zglf,*Δ*poxB*::*acs*,Δ*pfkA,*Δ*pykF*	This study
R07	*E. coli* BW25113,Δ*ptsG*::*glk,*Δ*galR*::*zglf,*Δ*poxB*::*acs*,Δ*pgi,*Δ*zwf*	This study
R08	*E. coli* BW25113,Δ*ptsG*::*glk,*Δ*galR*::*zglf,*Δ*poxB*::*acs*,Δ*pgi*,RBSL1-zwf	This study
R09	*E. coli* BW25113,ΔptsG::*glk*,Δ*galR*::*zglf*,Δ*poxB*::*acs*,Δ*pgi*,RBSL2-zwf	This study
R10	*E. coli* BW25113,ΔptsG::*glk*,Δ*galR*::*zglf*,Δ*poxB*::*acs*,Δ*pgi*,RBSL3-zwf	This study
R11	*E. coli* BW25113,ΔptsG::*glk*,Δ*galR*::*zglf*,Δ*poxB*::*acs*,Δ*pgi*,RBSL4-zwf	This study
R12	*E. coli* BW25113,ΔptsG::*glk*,Δ*galR*::*zglf*,Δ*poxB*::*acs*,Δ*pgi*,RBSL5-zwf	This study
R13	*E. coli* BW25113,Δ*ptsG*::*glk,*Δ*galR*::*zglf,*Δ*poxB*::*acs*,Δ*pgi*,119 Promoter-zwf	This study
R14	*E. coli* BW25113,ΔptsG::*glk*,Δ*galR*::*zglf*,Δ*poxB*::*acs*,Δ*pgi*,Δ*pgm*	This study
R15	*E. coli* BW25113,ΔptsG::*glk*,Δ*galR*::*zglf*,Δ*poxB*::*acs*,Δ*pgi*,Δ*pgm*,RBSL5-zwf	This study
Plasmids		
pYB1s	p15A ori, pBAD promoter, Str^R^	Laboratory
pLB1a	R6K ori, pBAD promoter, Amp^R^	Laboratory
p01	pYB1s containing TbIPS gene from *Trypanosoma brucei*	Laboratory
p02	pLB1a containing EcIMP gene from *E.coli*	Laboratory
p03	pLB1a containing ScIPS gene from *saccharomyces cerevisiae*	Laboratory
pR01	pYB1s containing TbIPS-EcIMP genes	This study
pR02	pYB1s containing EcIMP-TbIPS genes	This study
pR03	pYB1s containing TbIPS-ScIPS-EcIMP genes	This study

### Culture conditions

For expression of proteins, strains were cultured in LB medium with appropriate antibiotic (streptomycin 50 mg/L) at 37 °C and 220 rpm. The strains were incubated at 30 °C and 220 rpm and induced by adding l-arabinose (2 g/L). For high-density fermentation of recombinant strains, strains were initially cultured in LB medium at 37 °C and 220 rpm to produce a seed culture. Then, the seed culture was inoculated into 500 mL modified inorganic salts medium in a 1-L fermenter (Shanghai Bailun Biotechnology Co., Ltd.). The fermentation was conducted at 37 °C and pH 7.0, and initially 10 g/L glucose and 10 g/L glycerol were added. The dissolved oxygen (DO) level was maintained at 30–50% (v/v) by air flow at 1 L/min and changing the agitation speed from 500 to 1100 rpm. Feeding was started when the initial glucose concentration decreased below 1 g/L and was adjusted to maintain the residual glucose level below 1 g/L. The cells were induced by adding l-arabinose (2 g/L) at 30 °C when strains grew to suitable biomass (OD_600_ = 30).

### Bioconversion conditions

The recombinant strains were induced and harvested, then suspended in a bioconversion mixture containing 50 mM glucose. The bioconversions were performed for 10 h at 37 °C and 220 rpm in 1 × M9 salts buffer (Na_2_HPO_4_·7H_2_O 12.8 g/L, KH_2_PO_4_ 3 g/L, NaCl 0.5 g/L, NH_4_Cl 1 g/L) containing 1 mM MgSO_4_. The cell densities (OD_600_) at the starting and end points of the bioconversions were all almost 30. For the scaled-up production of inositol, ex situ and in situ bioconversions were used for inositol production by strain R04. For ex situ bioconversions, induced cells were harvested by centrifugation and suspended in 500 mL buffer containing 1 × M9 salts with 1 mM MgSO_4_ in a 1-L fermenter. Cells (OD_600_ = 80) were incubated with 30–50% DO at 37 °C. When the bioconversion starts, glucose is added in batches. The bioconversion in situ in a 1-L fermenter was started when the cells grew to the density of OD_600_ = 80, glucose is added in batches. The supply from the alkali pump is changed from NH_3_·H_2_O to NaOH, resulting in the transformation of cell growth to bioconversion. Cells were incubated with 30–50% DO at 37 °C.

### Analytical methods

Cell density was estimated by measuring optical density at 600 nm with a spectrophotometer. Recombinant enzyme expression was compared and analyzed by SDS-PAGE. For the preparation of SDS-PAGE samples, induced cells were harvested and suspended in 50 mM phosphate buffer (pH 7.0) with cell density OD_600_ = 10. The cells were lysed by ultrasonic disruption. The mixture was centrifuged, and the supernatant was mixed with isometric 2× protein loading buffer. After boiling for 10 min, equal volumes of sample were loaded onto gels. Concentrations of glucose, glycerol and inositol in culture supernatant were measured by high-performance liquid chromatography (HPLC) with a Bio-Rad Aminex HPX-87 H column (7.8 × 300 mm; Hercules, CA, USA) and a refractive index detector. Samples taken from bioconversions were centrifuged, and HPLC samples were obtained by filtration–sterilization of the supernatants. The analysis was performed with a flow rate of 0.5 mL/min using 5 mM H_2_SO_4_ as the mobile phase at 40 °C. The retention times of G-6-P, glucose and inositol were 7.621, 10.633, and 11.188 min respectively (Additional file 1: Fig. S5).

## Supplementary information

**Additional file 1: Table S1** Primers used in this study. **Fig. S1.** SDS-PAGE for expressions of IPS and IMP by optimizing plasmid expression systems. **Fig. S2.** The expressions of key enzymes of engineered strain R04 in 1-L fermenter for high-density fermentation. **Fig. S3.** Inositol production by strains R12, R14 and R15. **a** SDS-PAGE showing the expression of IPS (approximately 60 kDa) and IMP (approximately 30 kDa). **b** Production of inositol by strains R14, R12 and R15 cultivated in high-density fermentation and in shaken flasks, respectively. The columns correspond to “concentration of inositol”, and the lines and symbols correspond to “stoichiometric yield”. The recombinant strains were induced and harvested, then suspended in a bioconversion mixture containing 50 mM glucose. The bioconversions were performed at 37 °C and 220 rpm for 10 h. **Fig. S4.** Comparison of strains R01 and R04. **a** Production of inositol in strains R01 and R04 transformed with plasmids p01 and p02. The recombinant strains were induced and harvested, then suspended in a bioconversion mixture containing 50 mM glucose. The bioconversions were performed at 37 °C and 220 rpm for 10 h. **b** High-density fermentation of strains R01 and R04. Cells were cultured in LB medium at 37 °C. Then, this seed solution was inoculated into 500 mL inorganic medium with glycerol and glucose as mixed carbon source in a 1-L fermenter. **Fig. S5.** HPLC chromatograms of G-6P, glucose and inositol. The x-axis shows retention time and the y-axis the refractive index detector (RID) signal.

## Data Availability

Not applicable.

## Abbreviations

OD_600_: The optical density of cells at 600 nm
IPS: Inositol 1-phosphate synthase
IMP: Inositol monophosphatase
G-6-P: Glucose-6-phosphate
PPP: Pentose phosphate pathway
RBS: Ribosome binding site
pBAD: An araBAD promoter
SDS-PAGE: Sodium dodecyl sulfate–polyacrylamide gel electrophoresis
HPLC: High-performance liquid chromatography
