# Supplementary material for: Efficient production of myo-inositol in Escherichia coli through metabolic engineering
Source: Microb Cell Fact. 2020 May 24;19:109. doi: 10.1186/s12934-020-01366-5 (PMC7247202; doi:10.1186/s12934-020-01366-5)

**Additional file 1**

**Table S1** Primers used in this study.

| Primers | Sequence (5’-3’) |
| --- | --- |
| **inositol-synthetic pathway** |  |
| TbIPS-F | GCGCGGCAGCCTCGAGATGCCGGCAGTGCGTACGAA |
| TbIPS-R | CCGAGCTCACCACTAGTTTAGCTGCCTACGCCACGC |
| EcIMP-F | GCGCGGCAGCCTCGAGATGCATCCGATGCTGAACATCGCC |
| EcIMP-R | CCGAGCTCACCACTAGTTTAACGCTTCAGAGCGTCGC |
| **gene deletion** |  |
| *Δpgi*-up-F | cattttcagccttggcacaa |
| *Δpgi*-up-R | GGCCTACATATCGACGATGATAGCAATACTCTTCTGATTT |
| *Δpgi*-down-F | aaatcagaagagtattgctatcatcgtcgatatgtaggcc |
| *Δpgi*-down-R | GGCAAAAATGCCATACAGAAC |
| *ΔpfkA*-up-F | TATATAGCGCGTTACGCATG |
| *ΔpfkA*-up-R | TCCGAAATCAGACTACCTCTGAACTTTGGAATGCAAAATG |
| *ΔpfkA*-down-F | AGAGGTAGTCTGATTTCGGAAAAAGGCAGATTCCTTTACC |
| *ΔpfkA*-down-R | GTGACTGACGAATCACCACG |
| *ΔpykF*-up-F | TCATGCCAACTATCAGCATA |
| *ΔpykF*-up-R | CACAAAAGCAATAGACAGTCTTAGTCTTTAAGTTGAGAAGGATGGGAG |
| *ΔpykF*-down-F | GACTAAGACTGTCTATTGCTTTTGTGAATTAATTTGTATATCGAAGCGCCCTG |
| *ΔpykF*-down-R | GAGCTGCGTCATCTTTAGCA |
| *Δzwf*-up-F | agaaacgattcaccgtcggt |
| *Δzwf*-up-R | ATAAAGGATAAGCGCAGATAGTCATTCTCCTTAAGTTAAC |
| *Δzwf*-down-F | gttaacttaaggagaatgactatctgcgcttatcctttat |
| *Δzwf*-down-R | CTGGATAGTGTTCATAAGGC |
| *Δpgm*-up-F | atccgacattttacggcgta |
| *Δpgm*-up-R | tacgcgtttttcagaacttccattgctttgtcctttgtct |
| *Δpgm*-down-F | agacaaaggacaaagcaatggaagttctgaaaaacgcgta |
| *Δpgm*-down-R | gttctttttcgttcccgcct |
| ***zwf* regulation** |  |
| *zwf*-RBSL1-up-F | GATGTAAAGAGACTCACGGGTAATG |
| *zwf*-RBSL1-up-R | GAGAAGCCTTTGGAACTTGGTACGACAAGTTAACTAACCCGGTAC |
| *zwf*-RBSL1-down-F | GTCGTACCAAGTTCCAAAGGCTTCTCATGGCGGTAACGCAAACAGC |
| *zwf*-RBSL1-down-R | TCCAGCATCGCGCCGAGACGGCTGAATG |
| *zwf*-RBSL2-up-F | GATGTAAAGAGACTCACGGGTAATG |
| *zwf*-RBSL2-up-R | GGTGCTGTAATAAAGGTGCTGGTGCAAGTTAACTAACCCGGTAC |
| *zwf*-RBSL2-down-F | GCACCAGCACCTTTATTACAGCACCATGGCGGTAACGCAAACAGC |
| *zwf*-RBSL2-down-R | TCCAGCATCGCGCCGAGACGGCTGAATG |
| *zwf*-RBSL3-up-F | GATGTAAAGAGACTCACGGGTAATG |
| *zwf*-RBSL3-up-R | AGGGTCGGCTTGCTTATTATACTCCGAAGTTAACTAACCCGGTAC |
| *zwf*-RBSL3-down-F | CGGAGTATAATAAGCAAGCCGACCCTATGGCGGTAACGCAAACAGC |
| *zwf*-RBSL3-down-R | TCCAGCATCGCGCCGAGACGGCTGAATG |
| *zwf*-RBSL4-up-F | GATGTAAAGAGACTCACGGGTAATG |
| *zwf*-RBSL4-up-R | TGGCTTATCCTAGATACGTGAGCGTAAGTTAACTAACCCGGTAC |
| *zwf*-RBSL4-down-F | ACGCTCACGTATCTAGGATAAGCCAATGGCGGTAACGCAAACAGC |
| *zwf*-RBSL4-down-R | TCCAGCATCGCGCCGAGACGGCTGAATG |
| *zwf*-RBSL5-up-F | GATGTAAAGAGACTCACGGGTAATG |
| *zwf*-RBSL5-up-R | GACTATTCCCCTGAAATGGTAAGAAAGTTAACTAACCCGGTAC |
| *zwf*-RBSL5-down-F | TCTTACCATTTCAGGGGAATAGTCATGGCGGTAACGCAAACAGC |
| *zwf*-RBSL5-down-R | TCCAGCATCGCGCCGAGACGGCTGAATG |

**Fig. S1** SDS-PAGE for expressions of IPS and IMP by optimizing plasmid expression systems.


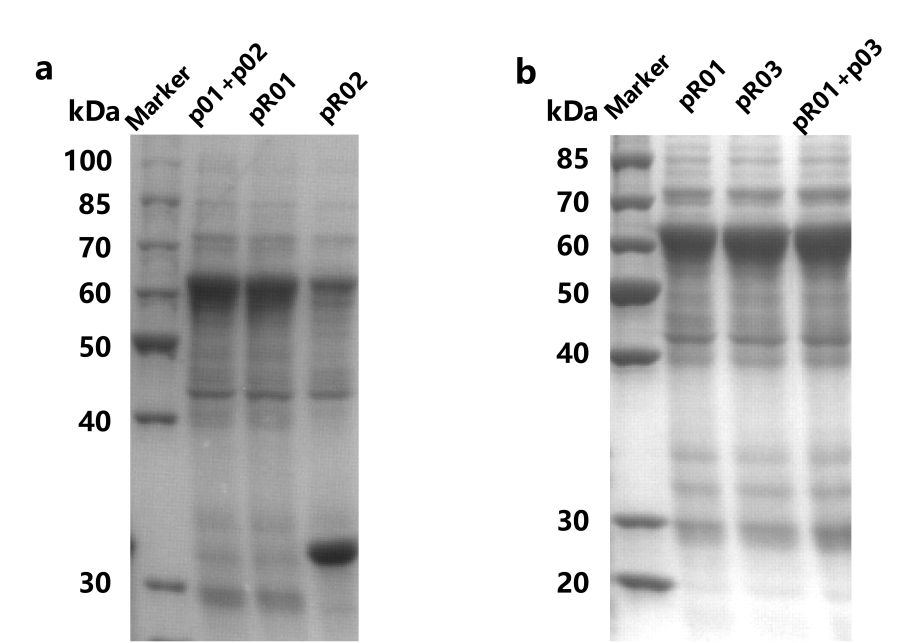


**Fig. S2** The expressions of key enzymes of engineered strain R04 in 1-L fermenter for high-density fermentation


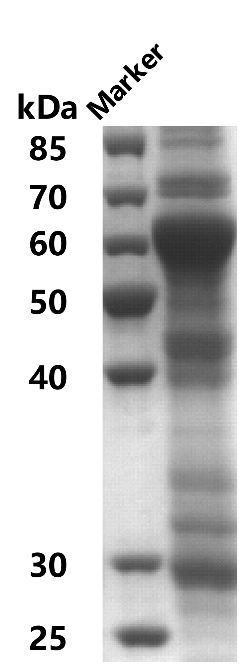


**Fig. S3** Inositol production by strains R12, R14 and R15. **a** SDS-PAGE showing the expression of IPS (approximately 60 kDa) and IMP (approximately 30 kDa). **b** Production of inositol by strains R14, R12 and R15 cultivated in high-density fermentation and in shaken flasks, respectively. The columns correspond to “concentration of inositol”, and the lines and symbols correspond to “stoichiometric yield”. The recombinant strains were induced and harvested, then suspended in a bioconversion mixture containing 50 mM glucose. The bioconversions were performed at 37 °C and 220 rpm for 10 h.


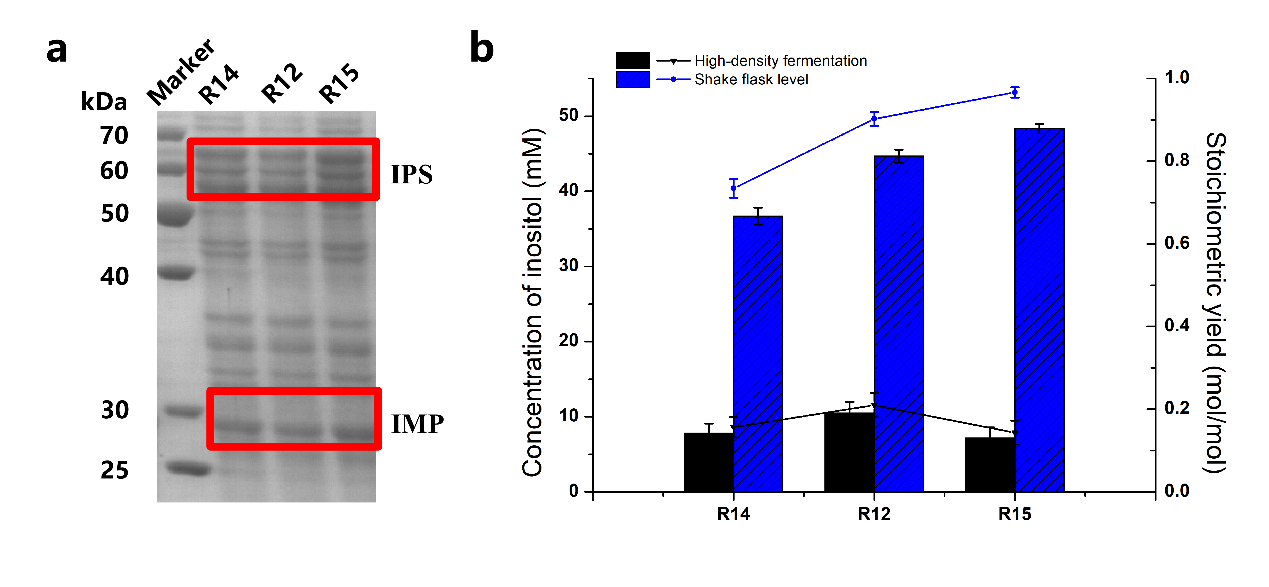


**Fig. S4** Comparison of strains R01 and R04. **a** Production of inositol in strains R01 and R04 transformed with plasmids p01 and p02. The recombinant strains were induced and harvested, then suspended in a bioconversion mixture containing 50 mM glucose. The bioconversions were performed at 37 °C and 220 rpm for 10 h. **b** High-density fermentation of strains R01 and R04. Cells were cultured in LB medium at 37 °C. Then, this seed solution was inoculated into 500 mL inorganic medium with glycerol and glucose as mixed carbon source in a 1-L fermenter.


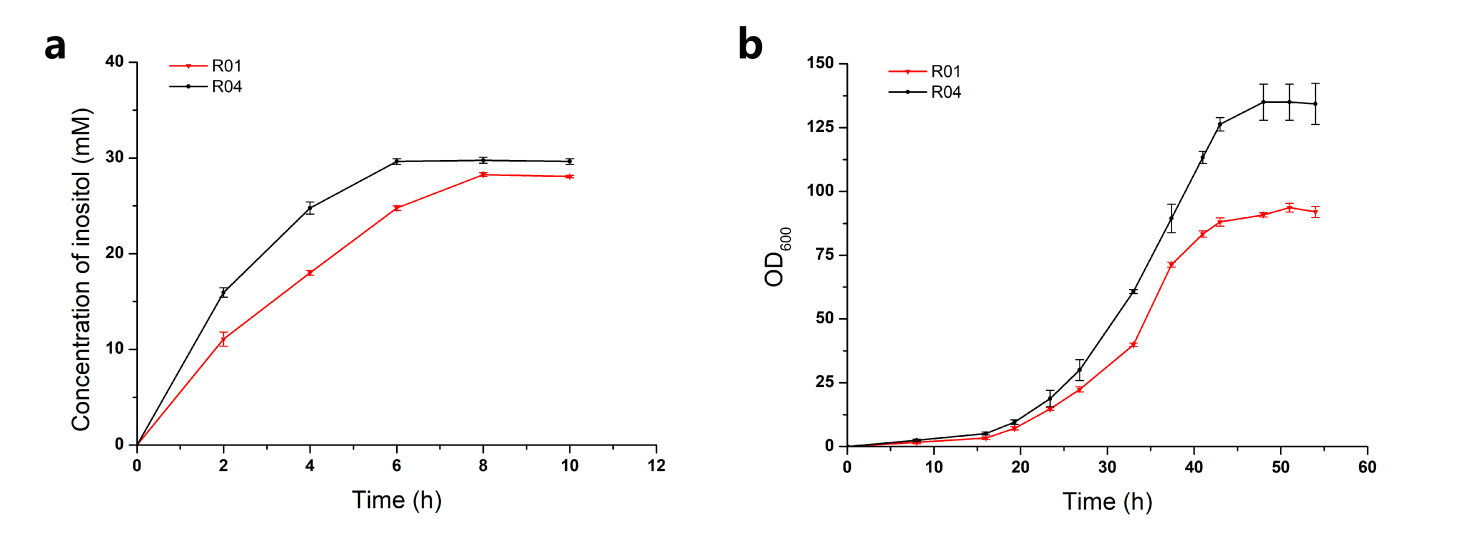


**Fig. S5** HPLC chromatograms of G-6P, glucose and inositol. The x-axis shows retention time and the y-axis the refractive index detector (RID) signal.


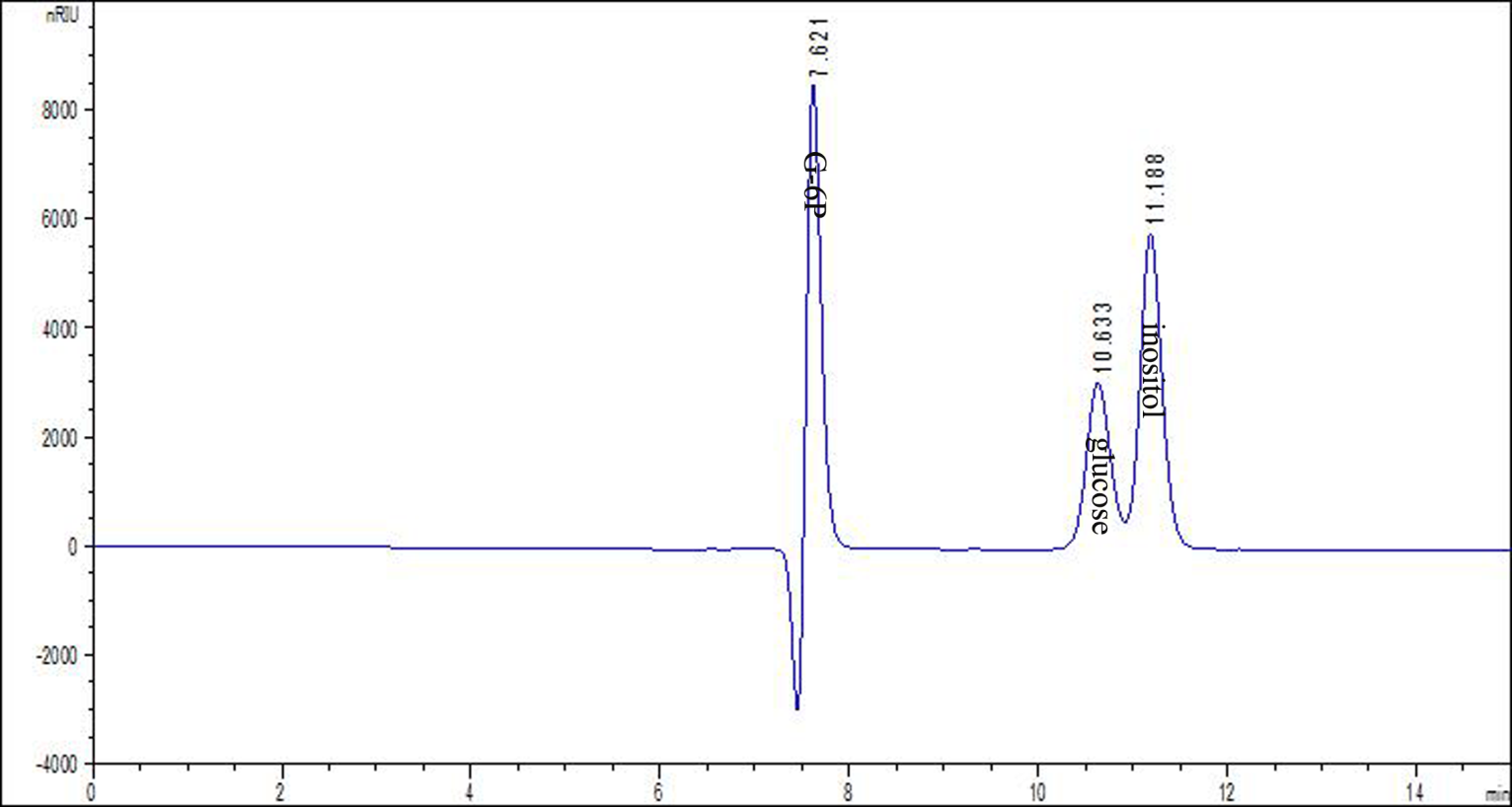

Supplement: Supplementary file 1 — Additional file 1: Table S1 Primers used in this study. Fig. S1. SDS-PAGE for expressions of IPS and IMP by optimizing plasmid expression systems. Fig. S2. The expressions of key enzymes of engineered strain R04 in 1-L fermenter for high-density fermentation. Fig. S3. Inositol production by strains R12, R14 and R15. a SDS-PAGE showing the expression of IPS (approximately 60 kDa) and IMP (approximately 30 kDa). b Production of inositol by strains R14, R12 and R15 cultivated in high-density fermentation and in shaken flasks, respectively. The columns correspond to “concentration of inositol”, and the lines and symbols correspond to “stoichiometric yield”. The recombinant strains were induced and harvested, then suspended in a bioconversion mixture containing 50 mM glucose. The bioconversions were performed at 37 °C and 220 rpm for 10 h. Fig. S4. Comparison of strains R01 and R04. a Production of inositol in strains R01 and R04 transformed with plasmids p01 and p02. The recombinant strains were induced and harvested, then suspended in a bioconversion mixture containing 50 mM glucose. The bioconversions were performed at 37 °C and 220 rpm for 10 h. b High-density fermentation of strains R01 and R04. Cells were cultured in LB medium at 37 °C. Then, this seed solution was inoculated into 500 mL inorganic medium with glycerol and glucose as mixed carbon source in a 1-L fermenter. Fig. S5. HPLC chromatograms of G-6P, glucose and inositol. The x-axis shows retention time and the y-axis the refractive index detector (RID) signal. [file 12934_2020_1366_MOESM1_ESM.docx]
